# Análisis Coste-Efectividad de una Unidad de ICTUS: Estudio del Caso del Complejo Hospitalario Universitario de Santiago de Compostela

**DOI:** 10.31083/RN39320

**Published:** 2025-03-27

**Authors:** Francisco Reyes-Santias, Alicia do Carme Pastoriza Castro, Maria Santamaria-Cadavid, Emilio Castro, Manuel Rodriguez-Yañez, Jose Maria Prieto-Gonzalez, Beatriz Aibar-Guzman

**Affiliations:** ^1^GEN (Governance and Economics research Network), Departamento de Organización de Empresas y Marketing Universidad de Vigo, 32004 Ourense, España; ^2^FIDIS (Fundación Instituto de Investigación Sanitaria), Hospital Clínico Santiago de Compostela, 15706 Santiago de Compostela, España; ^3^Centro de Investigación Biomédica en Red Enfermedades Cardiovaculares (CIBERCV) Instituto de Salud Carlos III, Hospital Clínico Santiago de Compostela, 15706 Santiago de Compostela, España; ^4^Departamento de Economía Financiera y Contabilidad, Universidad de Santiago de Compostela (USC), 15782 Santiago de Compostela, España; ^5^Servicio de Neurología, Hospital Clínico de Santiago de Compostela (CHUS), Servicio Galego da Saúde (SERGAS), 15706 Santiago de Compostela, España

**Keywords:** ictus, hospital, costes, coste-efectividad, stroke, hospital, costs, cost-effectiveness

## Abstract

**Introducción:**

el ictus tiene un impacto enorme en la sociedad, tanto desde el punto de vista social como económico. Es la causa más habitual de ingreso y prolongación de la estancia en la planta de neurología.

**Métodos:**

En este trabajo se realiza un análisis coste-efectividad del tratamiento del ictus en el Complejo Hospitalario de Santiago de Compostela, donde se trata a los enfermos en una unidad de ictus. En primer lugar, se lleva a cabo un análisis de los costes del tratamiento en la unidad de ictus que no se había realizado hasta el momento. Los costes se comparan con los que habría si se siguiera tratando la enfermedad sin unidad de ictus, verificando que la existencia de dicha unidad implica un incremento de los costes del tratamiento. A continuación, se analizan diversos parámetros que reflejan la eficacia del tratamiento para finalmente realizar un análisis coste-efectividad con el objetivo de determinar si el aumento de los costes está justificado por una mejora en los resultados.

**Resultados:**

el tratamiento del ictus en la unidad centralizada de ictus sería coste-efectivo si tomamos como medida de efectividad la mortalidad durante la hospitalización o a los tres meses del alta, el parámetro mRS en el momento del alta o a los tres meses del alta y la discapacidad grave en el momento del alta.

**Conclusión:**

se puede afirmar que el incremento de los costes de una unidad de ictus se ve justificado por la mejora en la salud de los pacientes.

## Abstract (en)

**Introduction:**

Stroke has an enormous impact on society, both socially and economically. It is the most common cause of admission and prolonged stay in the Neurology ward.

**Methods:**

We performed a cost-effectiveness analysis of stroke treatment in the Complejo Hospitalario de Santiago de Compostela, where patients are treated in a stroke unit. Firstly, an analysis of the costs of treatment in the stroke unit was carried out, which had not been done before. The costs were compared with those that would be incurred if the disease was treated without the stroke unit, verifying that the existence of such a unit implies an increase in treatment costs. Next, various parameters reflecting the efficacy of the treatment were analyzed to finally perform a cost-effectiveness analysis in order to determine whether the increase in costs is justified by an improvement in outcomes.

**Results:**

Stroke treatment in the centralized stroke unit would be cost-effective if we use mortality during hospitalization or 3 months after discharge, the mRS parameter at discharge or 3 months after discharge, and severe disability at discharge as measures of effectiveness.

**Conclusion:**

The increased costs of a stroke unit are justified by the improved health of the patients.

## 1. Introducción

En un contexto en el que el gasto sanitario está en constante aumento es necesario dotarse de herramientas que permitan asignar eficientemente los recursos disponibles. Es por eso que es cada vez más frecuente la evaluación económica de tecnologías sanitarias, puesto que nos aporta información útil a la hora de tomar decisiones en el ámbito clínico [1]. Los distintos métodos de evaluación económica nos permiten comparar tratamientos o programas alternativos y elegir cuál es el mejor, en función de sus costes y de sus resultados [2].

Por otro lado, el ictus tiene un impacto enorme en la sociedad, tanto desde el punto de vista social como económico [3, 4, 5]. Es la causa más habitual de ingreso y prolongación de la estancia en la planta de neurología [6]. Entre las distintas formas de abordar esta enfermedad, se demostró que las unidades de ictus son la mejor alternativa [7, 8]. De hecho, desde 1996 se fijó como objetivo que todos los afectados por ictus de la Unión Europea fuesen atendidos en unidades de ictus [9]. En Galicia, son cinco los hospitales que cuentan con una unidad de ictus: la unidad más antigua en su creación ha sido la del Complejo Hospitalario Universitario de Santiago de Compostela (C.H.U.S), y posteriormente el Complejo Hospitalario Universitario de A Coruña (C.H.U.A.C), el Hospital Álvaro Cunqueiro de Vigo, y más recientemente el Hospital Lucus Augusti de Lugo y el Complejo Hospitalario Universitario deOurense (C.H.O.U.) también disponen de unidad de ictus. El objetivo de este estudio es realizar un análisis coste-efectividad del tratamiento y cuidado de los enfermos de ictus en el C.H.U.S durante el primer semestre del año 2022.

## 2. Métodos

Para llevar a cabo a análisis se utilizó información de los pacientes ingresados por ictus C.H.U.S. antes y después de la centralización de la Unidad de Ictus en un mismo espacio físico y su monitarización. Los datos se corresponden con el semestre previo a la centralización de la Unidad de Ictus (de enero a junio de 2021) y con el primer semestre de 2022. Contamos con la información sobre la situación de los pacientes en el momento del ingreso y con indicadores de la efectividad en el momento del alta y a los tres meses del alta.

Desde 2004 existe en el C.H.U.S. una Unidad de Ictus repartida en dos habitaciones de dos camas cada una en la planta de hospitalización de Neurología. Desde julio de 2021, se ha centralizado la Unidad de Ictus como un área de hospitalización específica, constituida por seis camas con un control de enfermería propio, con monitorización centralizada y obtención de la información clínica por parte de los facultativos en tiempo real, situada en la planta de Neurología del hospital, a cargo de personal de enfermería con formación y experiencia en el manejo del ictus agudo y sus complicaciones. Denominamos, a efectos de este trabajo y para especificar los cambios en la estructura física de las dos Unidades de Ictus diferenciadas en el tiempo como Unidad “descentralizada” de Ictus a la puesta en funcionamiento en 2004 y Unidad centralizada de Ictus a la puesta en funcionamiento a partir de la segunda mitad del año 2021.

Para la determinación de los costes sanitarios, se cuantificaron los distintos componentes de coste del tratamiento en la unidad centralizada de ictus y en la unidad “descentralizada” de ictus a fin de comparar el coste actual y el coste que habría si se siguiese tratando a los pacientes como se hacía anteriormente (en la unidad “descentralizada” de ictus). Para ello, en primer lugar, es necesario identificar cuáles son los recursos consumidos en el desarrollo de los procesos objeto de estudio, para luego cuantificar dicho consumo en unidades físicas y, finalmente, proceder a su valoración en unidades monetarias [10]. Diferenciaremos entre costes de personal y costes de funcionamiento.

### 2.1 Costes de Personal

Para su determinación es necesario conocer el tiempo que cada uno de los profesionales sanitarios implicados dedica al tratamiento y cuidado de los pacientes con ictus, tanto en la situación inicial (unidad “descentralizada” de ictus) como después de la creación de la unidad centralizada de ictus en el C.H.U.S y el coste/hora de cada categoría de profesional sanitario. La puesta en marcha de la Unidad específica de Ictus en el hospital ha conllevado la adscripción a dicha Unidad de una Técnico Auxiliar de Enfermería y una enfermera a tiempo completo. Del mismo modo, mientras antes de la Unidad centralizada las guardias se organizaban con un residente o un médico adjunto de presencia física y otro médico adjunto de guardia requerida, con la nueva Unidad se han reorganizado las guardias con dos facultativos de presencia física.

A partir del salario bruto anual, el plus de guardia de los médicos y la jornada anual se ha calculado el coste por hora correspondiente a cada categoría profesional. Una vez calculado este coste se multiplica por las horas diarias de trabajo para determinar el coste diario, a partir del cual se calcula el coste semestral. En el caso de los costes indirectos, su imputación se realiza utilizando como clave de distribución el número de camas. El resultado se muestra en la siguiente tabla (Tabla 1).

**Tabla 1.** **Comparación de los costes de personal por categorías profesionales**.

Categoría profesional		UNIDAD DESCENTRALIZADA DE ICTUS	UNIDAD CENTRALIZADA DE ICTUS
Médico		21.367 €	29.958 €
Enfermero		28.849 €	108.166 €
Auxiliar		13.387 €	66.941 €
Celador		7.464 €	12.444 €
Total		71.067 €	217.509 €
Comparación de los costes de equipamiento			
		UNIDAD DESCENTRALIZADA DE ICTUS	UNIDAD CENTRALIZADA DE ICTUS
Valor de los equipos y otros bienes		52.236 €	90.638 €
Coste de amortización semestral		3.062 €	5.313 €
Coste de mantenimiento semestral		196 €	340 €
Coste total de equipamiento semestral		3.258 €	5.653 €
Comparación de los costes generales			
CONCEPTOS		UNIDAD DESCENTRALIZADA DE ICTUS	UNIDAD CENTRALIZADA DE ICTUS
Limpieza		1.032 €	1.548 €
Seguridad		110 €	164 €
Suministros		374 €	561 €
Vestuario		31 €	47 €
Edificios y otras construcciones		92 €	137 €
Maquinaria, instalaciones y utensilios		272 €	407 €
Otros		46 €	68 €
Hostelería		4.640 €	5.141 €
Total		6.597 €	8.673 €
Comparación de los costes fungibles			
MATERIAL		UNIDAD DESCENTRALIZADA DE ICTUS	UNIDAD CENTRALIZADA DE ICTUS
Material sanitario		4.578 €	4.878 €
Fármacos		65.909 €	72.806 €
Coste total de los fungibles		70.487 €	77.684 €
Comparación de los costes totales			
CONCEPTO		IMPORTE	
		UNIDAD DESCENTRALIZADA DE ICTUS	UNIDAD CENTRALIZADA DE ICTUS
Costes de personal		71.067 €	217.508 €
Costes de funcionamiento	Costes de equipamiento	3.258 €	5.653 €
	Costes generales	6.597 €	8.075 €
	Costes de fungibles	70.487 €	77.684 €
Costes totales		151.409 €	308.920 €
Coste por paciente		515 €	1.251 €

El número medio de pacientes se calculó teniendo en cuenta que los pacientes ingresados durante el primer semestre de 2021 (antes de la creación de la unidad de ictus) y durante el primer semestre de 2022 (después de la creación de la unidad de ictus) fueron 340 y 247, respectivamente. Tasa de cambio euros a dólares: 1 € = 1,03$. Fuente: Elaboración propia.

### 2.2 Costes de Funcionamiento

Los costes de funcionamiento comprenden tres categorías: costes de equipamiento, costes generales y costes de fungibles.

Dentro de los costes de equipamiento se distingue entre costes de amortización y costes de mantenimiento. Para el cálculo del coste de amortización, siguiendo a Drummond *et al*. (2001) [11], se utiliza el método del coste anual equivalente, ya que permite comparar un único año de datos de coste para cada alternativa. La fórmula utilizada es la siguiente: CAUE = Coste histórico x $\frac{{\left(1+i\right)}^{n}\,x\,i}{{\left(1+i\right)}^{n}-1}$, donde “i” es la tasa de interés y “n” la vida útil estimada. Siguiendo a Gomez-Ulla *et al*. (2008) [12], como tasa de interés se ha utilizado el 3%, por ser la tasa recomendada por los analistas, y como la vida estimada se han considerado 10 años.

En lo relativo al coste de mantenimiento de los equipos y de otros bienes, de acuerdo con Alonso Alperi (2006) [13], en el caso concreto del C.H.U.S, este coste se sitúa entre un 7% y un 8% del valor de la inversión, por lo que, siguiendo a esta autora, utilizaremos un 7.5%, la media de los dos valores (Tabla 1).

Los costes generales se calculan a partir de los importes recogidos en los presupuestos de Galicia correspondientes al ejercicio 2022, que recogen los gastos totales del área sanitaria de Santiago de Compostela y Barbanza. Como el período de estudio es el semestre, estas cantidades se dividen entre dos. Para repartirlos se utiliza como clave de distribución los metros cuadrados de superficie del área sanitaria de Santiago de Compostela y Barbanza (Tabla 1).

Los fungibles son los materiales sanitarios y fármacos consumidos por los pacientes con ictus durante su estancia en el hospital. La determinación de este coste se realizó a partir de datos proporcionados por el servicio de farmacia del C.H.U.S (Tabla 1).

### 2.3 Costes Totales y Costes Por Paciente

La siguiente tabla (Tabla 1) resume todos los datos y determina el coste semestral del tratamiento del ictus con cada alternativa.

## 3. Efectividad de los Tratamientos 

De acuerdo con Langhorne y Ramachandra (2020) [14], como medidas de efectividad se eligieron la mortalidad durante el ingreso y a los tres meses, la discapacidad grave en el momento del alta y a los tres meses y el parámetro La escala de Rankin modificada (mRS-Modified Rankin Scale) en el momento del alta y a los tres meses. En la siguiente tabla (Tabla 2, Ref. [15, 16]) se muestran los valores de dichas medidas. En el caso de la situación inicial (unidad “descentralizada” de ictus) se tomaron los datos correspondientes al primer semestre de 2021, es decir, el semestre previo a la puesta en funcionamiento de la unidad centralizada de ictus en el C.H.U.S.

**Tabla 2.** **Comparación de las medidas de efectividad**.

Medida de Efectividad	Unidad Descentralizada de Ictus	Unidad Centralizada de Ictus
Mortalidad durante el ingreso	9.8%	6.1%
Mortalidad a los tres meses	16.7%	14.6%
Discapacidad grave en el alta	11.7%	8.1%
Discapacidad grave a los tres meses	0.9%	2.1%
mRS ≤2 en el alta	37.6%	46.2%
mRS ≤2 a los tres meses	53%	56.1%

La escala de Rankin modificada (mRS) es una escala para la valoración funcional de los afectados por patologías cerebrovasculares (Fernández *et al*., 2022) [15]. Es una de las medidas de resultado más utilizadas en estudios sobre el ictus (Duncan *et al*., 2000) [16]. Se considera que el pronóstico funcional de os pacientes es bueno cando el parámetro mRS es inferior o igual a 2. Fuente: Elaboración propia.

De acuerdo con los valores de las medidas de efectividad, excepto en el caso de la discapacidad grave a los tres meses, se observa una mejora de los resultados del tratamiento del ictus cuando se trata a los pacientes en la unidad de ictus.

En cuanto a la mortalidad durante la hospitalización, ésta es más elevada cuando se trata la enfermedad en la unidad descentralizada de ictus. En concreto, la mortalidad durante el ingreso se redujo en 3,7 puntos porcentuales desde que se dispone de la unidad de ictus:

∙ Mortalidad durante la hospitalización en la unidad descentralizada de ictus: 9,8%

∙ Mortalidad durante la hospitalización con unidad centralizada de ictus: 6,1%

La mortalidad a los tres meses también es inferior en los pacientes tratados en la unidad centralizada de ictus. En concreto, se reduce en 2,1 puntos porcentuales:

∙ Mortalidad a los tres meses unidad descentralizada de ictus: 16,7%

∙ Mortalidad a los tres meses con unidad centralizada de ictus: 14,6%

Por su parte, la escala de Rankin modificada es una de las medidas de resultado más empleadas en estudios sobre el ictus [16]. Se considera que el pronóstico funcional de los pacientes es bueno cuando el parámetro mRS es inferior o igual a 2. Como se muestra en la Tabla 2, es mayor el porcentaje de pacientes que presenta un mRS inferior o igual a 2 cuando se trata la enfermedad en la unidad centralizada de ictus, tanto en el momento del alta como a los tres meses. Por lo tanto, se puede afirmar que tratar a los pacientes en la unidad centralizada de ictus mejora su pronóstico funcional.

En lo relativo a la discapacidad, el porcentaje de pacientes que en el momento del alta están afectados por una discapacidad grave se reduce tras la puesta en marcha de la unidad centralizada de ictus:

∙ Discapacidad grave en el momento del alta unidad descentralizada de ictus: 11,7%

∙ Discapacidad grave en el mometno del alta con unidad centralizada de ictus: 8,1%

En cambio, a los tres meses a porcentaje de pacientes afectados por una discapacidad grave aumenta tras la puesta en marcha de la unidad centralizada de ictus:

∙ Discapacidad grave a los tres meses del alta sin unidad descentralizada de ictus: 0,9%

∙ Discapacidad grave a los tres meses del alta con unidad centralizada de ictus: 2,1%

Por lo tanto, centrándonos en los indicadores anteriores, excepto en el caso de la discapacidad grave a los tres meses, se observa una mejora de los resultados del tratamiento del ictus cuando se trata a los pacientes en la unidad centralizada de ictus. Entendemos que el peor resultado que presenta el caso de la discapacidad grave a los tres meses, se debe a que, al darse en la Unidad centralizada de Ictus una disminución de la mortalidad, parte de estos pacientes que sobreviven, presentarían un peor dato de discapacidad grave a los tres meses, lo que no sucedía antes de la reforma de la Unidad de Ictus, dado que estos pacientes presentaban un ratio de mortalidad superior.

## 4. Análisis Coste-Efectividad del Tratamiento en la Unidad de Ictus

Para la realización del análisis coste-efectividad se calcula la ratio de coste-efectividad incremental (ICER) para cada una de las medidas de efectividad anteriores. Esta ratio se determina dividiendo la diferencia entre los costes por paciente de los dos tratamientos por la diferencia entre los resultados de los dos tratamientos (Tabla 3).

**Tabla 3.** **Cálculo del ICER para cada medida de efectividad**.

Medida de efectividad	ICER
Mortalidad durante la hospitalización	–14.480
Mortalidad a los 3 meses del alta	–25.512
mRS ≤2 en el momento del alta	5.953
mRS ≤2 a los 3 meses del alta	17.282
Discapacidad grave en el momento del alta	–14.882
Discapacidad grave a los 3 meses del alta	44.646

Fuente: Elaboración propia. ICER, Ratio Incremental Coste-Efectividad.

A la hora de interpretar los datos hay que tener en cuenta que, en nuestro caso, dos de las medidas de efectividad empleadas (mortalidad y discapacidad grave) son medidas negativas, es decir, cuanto mejor sea el resultado del tratamiento, menores serán estos indicadores. Esto justifica que el valor del ICER sea negativo en el caso de mortalidad (tanto en hospitalización como a los tres meses) y discapacidad grave en el momento del alta, puesto que mejoraron tras la implantación de la unidad de ictus. Por otra parte, en el caso de discapacidad grave a los tres meses, el valor positivo del ICER se debe la que este indicador empeoró.

Para determinar si el tratamiento en la unidad centralizada de ictus es coste-efectivo tomamos como referencia el umbral de coste-efectividad para la toma de decisiones sobre la financiación de una nueva terapia o una nueva prueba complementaria de diagnóstico: entre 30.000 € y 40.000 € [2]. Si el ICER se sitúa por encima de este umbral, no sería coste-efectivo. En el caso contrario, sí lo sería. Cabe destacar que esta cifra se emplea cómo referencia cuando se utiliza como indicador de efectividad los años de vida ajustados por calidad. En este estudio, ante la imposibilidad de determinarlos, optamos por emplear los parámetros indicados anteriormente.

Como se muestra en la Tabla 3, para los indicadores en los que se observa una mejora con la puesta en funcionamiento de la unidad centralizada de ictus, el ratio es inferior a 30.000 € en términos absolutos. Por lo tanto, el tratamiento del ictus en la unidad centralizada de ictus sería coste-efectivo si tomamos como medida de efectividad la mortalidad durante la hospitalización o a los tres meses del alta, el parámetro mRS en el momento del alta o a los tres meses del alta y la discapacidad grave en el momento del alta.

A La hora de interpretar los datos hay que tener en cuenta que, en nuestro caso, dos de las medidas de efectividad empleadas (mortalidad y discapacidad grave) son medidas negativas, es decir, cuanto mejor sea el resultado del tratamiento, menores son estos indicadores. Esto justifica que el valor del ICER sea negativo en el caso de mortalidad (tanto en hospitalización como a los tres meses) y discapacidad grave en el momento del alta, puesto que mejoraron tras la implantación de la unidad de ictus. Por otra parte, en el caso de discapacidad grave a los tres meses, el valor positivo del ICER se debe la que este indicador empeoró.

**Fig. 1.**
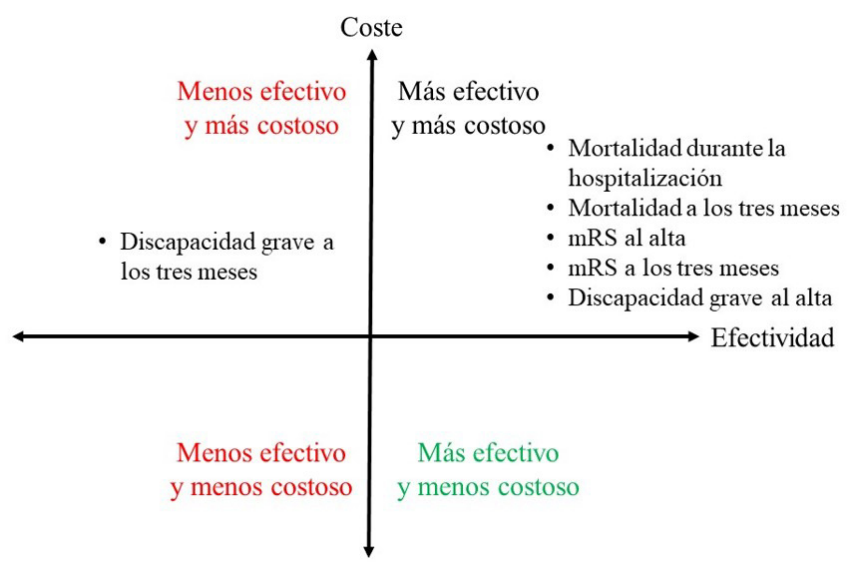
**Plano coste efectividad del tratamiento en unidad de ictus**.

Así, como se observa en la Fig. 1, de acuerdo con los resultados anteriores, si se toma como medida de efectividad de los cinco primeros indicadores utilizados, el tratamiento del ictus en la unidad centralizada de ictus se situaría en el primer cuadrante del plano coste-efectividad. En cambio, de elegir la discapacidad a los tres meses, esta alternativa se colocaría en el cuarto cuadrante.

## 5. Discusión

Nuestro estudio muestra que la unidad de ictus en el C.H.U.S implica un aumento de los costes del tratamiento de la enfermedad. Este resultado está en línea con los estudios llevados a cabo por Moodie *et al*. (2006) [17] en Australia y por Launois *et al*. (2004) [18] en Francia, en los que se compararon los costes de la unidad de ictus con los costes del tratamiento convencional.

Por otra parte, en nuestro estudio queda patente que la unidad de ictus es más eficaz en términos de reducción de la mortalidad, de mejora del pronóstico funcional y de reducción de la discapacidad en el momento del alta. Estos resultados son consistentes con los observados en la literatura. En concreto, el estudio realizado por Zhai *et al*. (2017) [19] mostró una reducción de la mortalidad durante el ingreso de casi 5 puntos porcentuales, así como una mejora en términos de mRS a los tres meses tras la creación de la unidad de ictus. Kalra *et al*. (2005) [20] también mostraron resultados similares: una reducción de la mortalidad a los tres meses de 6 puntos porcentuales y una mejora del mRS a los tres meses. Sin embargo, nuestros resultados en la efectividad son menores a los expuestos por Simal Hernández *et al*. (2022) [21] refiriendo una disminución de mortalidad y discapacidad del 20%. Nuestros resultados se encuentran en la línea de estudios como el de Morris *et al*. (2019) [22], con disminución de mortalidad en las Unidades centralizadas de Ictus, en el Reino Unido, del 1,8%.

En este sentido, Langhorne y Ramachandra (2020) [14], en un estudio de la Cochrane Library, han hallado pruebas de calidad moderada de que los pacientes con ictus que reciben atención hospitalaria organizada (unidad de ictus) tienen más probabilidades de estar independientes y viviendo en casa un año después del ictus. Los beneficios aparentes fueron independientes de la edad, el sexo, la gravedad inicial del ictus o el tipo de ictus, y fueron más evidentes en las unidades basadas en una sala de ictus diferenciada. No se observó un aumento sistemático de la duración de la estancia hospitalaria, pero estos hallazgos presentan una incertidumbre considerable.

Igualmente, y con resultados muy similares a los de nuestro estudio, para Wennman *et al*. (2023) [23], la tasa de mortalidad a 90 días fue del 12,9% para los casos ingresados en unidades hospitalarias de ictus y del 14,7% para los controles (ingresados en el hospital, pero no en unidades ad hoc).

## 6. Conclusión

La unidad centralizada de ictus presenta mejores resultados que la hospitalización convencional, en términos de mortalidad durante el ingreso y a los tres meses, en la valoración funcional de los afectados por patologías cerebrovasculares tanto en el momento del alta como a los tres meses y en términos de discapacidad grave en el momento del alta. Por lo tanto, se puede afirmar que el incremento de los costes se ve justificado por la mejora en la salud de los pacientes y el ratio coste-efectividad favorable al establecimiento y funcionamiento de la Unidad centralizada de Ictus.

## Disponibilidad de Datos y Materiales

Data is available upon request.

## Contribuciones de los Autores

FRS: conceptualización, metodología, validación, análisis formal, investigación, redacción-borrador original, redacción-revisión y edición, visualización, supervisión; ACPC: metodología, validación, análisis formal, investigación, redacción-borrador original, visualización; MSC: base de datos, validación, investigación, redacción-revisión y edición; EC: base de datos, validación, investigación, redacción-borrador original, redacción-revisión y edición; MRY: base de datos, validación, investigación, redacción-revisión y edición; JMPG: conceptualización, validación, investigación, redacción-revisión y edición, visualización, supervisión; BAG: conceptualización, metodología, validación, análisis formal, investigación, redacción-borrador original, redacción-revisión y edición, visualización, supervisión. Todos los autores leyeron y aprobaron el manuscrito final. Todos los autores participaron lo suficiente en el trabajo y aceptaron ser responsables de todos los aspectos del mismo.

## Aprobación Ética y Consentimiento Informado

Not applicable.

## Agradecimientos

No aplicable.

## Financiación

Esta investigación no recibió financiación externa.

## Conflicto de Intereses

Los autores declaran no tener conflictos de interés.
